# Organelles in *Blastocystis* that Blur the Distinction between Mitochondria and Hydrogenosomes

**DOI:** 10.1016/j.cub.2008.03.037

**Published:** 2008-04-22

**Authors:** Alexandra Stechmann, Karleigh Hamblin, Vicente Pérez-Brocal, Daniel Gaston, Gregory S. Richmond, Mark van der Giezen, C. Graham Clark, Andrew J. Roger

**Affiliations:** 1 Department of Biochemistry and Molecular Biology, Dalhousie University, Halifax, B3H 1X5, Canada; 2 School of Biological and Chemical Sciences, Queen Mary, University of London, London, E1 4NS, United Kingdom; 3 Department of Infectious and Tropical Diseases, London School of Hygiene and Tropical Medicine, London, WC1E 7HT, United Kingdom

**Keywords:** CELLBIO, EVO_ECOL

## Abstract

*Blastocystis* is a unicellular stramenopile of controversial pathogenicity in humans [1, 2]. Although it is a strict anaerobe, *Blastocystis* has mitochondrion-like organelles with cristae, a transmembrane potential and DNA [2–4]. An apparent lack of several typical mitochondrial pathways has led some to suggest that these organelles might be hydrogenosomes, anaerobic organelles related to mitochondria [5, 6]. We generated 12,767 expressed sequence tags (ESTs) from *Blastocystis* and identified 115 clusters that encode putative mitochondrial and hydrogenosomal proteins. Among these is the canonical hydrogenosomal protein iron-only [FeFe] hydrogenase that we show localizes to the organelles. The organelles also have mitochondrial characteristics, including pathways for amino acid metabolism, iron-sulfur cluster biogenesis, and an incomplete tricarboxylic acid cycle as well as a mitochondrial genome. Although complexes I and II of the electron transport chain (ETC) are present, we found no evidence for complexes III and IV or F_1_F_o_ ATPases. The *Blastocystis* organelles have metabolic properties of aerobic and anaerobic mitochondria and of hydrogenosomes [7, 8]. They are convergently similar to organelles recently described in the unrelated ciliate *Nyctotherus ovalis*[9]. These findings blur the boundaries between mitochondria, hydrogenosomes, and mitosomes, as currently defined, underscoring the disparate selective forces that shape these organelles in eukaryotes.

## Results and Discussion

### *Blastocystis* Has Proteins Characteristic of Hydrogenosomes

All “amitochondriate” protists and fungi studied to date have been shown to contain mitochondrion-derived double-membrane-bound organelles [10]. These organelles are typically classified into one of two types: anaerobic ATP-producing hydrogenosomes or mitosomes that have no obvious role in energy metabolism [5, 6, 10]. Two key enzymes of anaerobic energy metabolism in these organisms are pyruvate:ferredoxin oxidoreductase (PFO), which is responsible for the oxidative decarboxylation of pyruvate to form acetyl-CoA, and [FeFe] hydrogenase, which produces H_2_ gas [7]. These enzymes are located in the organelles of hydrogenosome-containing anaerobic protists and fungi but usually are found in the cytosol of those that have mitosomes.

**Figure 1:**
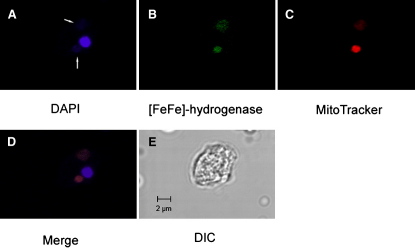
Confocal Immunofluorescence Microscopy Images Showing the Subcellular Localization of [FeFe] Hydrogenase in *Blastocystis* The images show a *Blastocystis* cell with one nucleus and two mitochondrion-like organelles. (A) DAPI staining of the nucleus (bright) and mitochondrial DNA (faint; see arrows); (B) immunolocalization of [FeFe] hydrogenase using anti-*Blastocystis*Hyd antibody; (C) MitoTracker orange staining of the mitochondrion-like organelles; (D) colocalization of MitoTracker orange, anti-*Blastocystis*Hyd antibody, and DAPI in the mitochondrion-like organelles; and (E) DIC image of the *Blastocystis* cell.

Among the *Blastocystis* ESTs we identified both a PFO and a “long-type” [FeFe] hydrogenase homolog. Phylogenies of both sequences indicate that they group with other eukaryote homologs from anaerobes or green algae (Figures S1 and S2). Both enzymes likely function in the *Blastocystis* mitochondrion-like organelles (MLOs) because both are predicted to have canonical mitochondrial-targeting peptides. These N-terminal extensions are enriched in hydrophobic and positively charged amino acids and form amphiphilic alpha helices [11] that are typically cleaved off after mitochondrial import (Table S1). To further test the predicted organellar location, we raised a homologous antibody against recombinant *Blastocystis* [FeFe] hydrogenase. Confocal fluorescence microscopy using this antibody showed that [FeFe] hydrogenase colocalizes with organelles that are stained by the transmembrane potential-dependent mitochondrion-specific dye MitoTracker (Figure 1 and Figure S3). Although at present we have no direct evidence of hydrogen production by the *Blastocystis* MLOs, the [FeFe] hydrogenase likely accepts electrons from PFO and produces molecular hydrogen, the defining characteristic of hydrogenosomes [7].

Interestingly, the [FeFe] hydrogenase has an additional C-terminal domain homologous to flavodoxins, a unique domain arrangement for this protein family of [FeFe] hydrogenases. Flavodoxins can be functionally interchangeable with ferredoxins and may be used as an alternative redox partner [12]. The flavodoxin domain may, thus, substitute for ferredoxin in transferring electrons from PFO to the [FeFe] hydrogenase domain in *Blastocystis*.

### *Blastocystis* Possesses a Mitochondrial Genome

**Figure 2:**
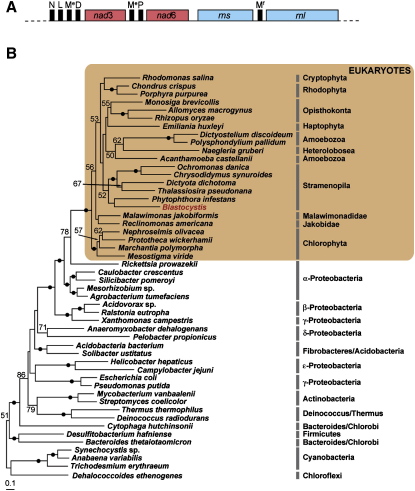
Analysis of the Organellar Genome Fragment (A) Schematic representation of an ∼6 kb fragment from the *Blastocystis* organellar genome. Black boxes represent tRNA genes, which are identified by the amino acid they incorporate. *nad*3 and *nad*6 are NADH dehydrogenase subunits. M^e^ is the elongator methionine tRNA, and M^f^ is the initiator (formyl-) methionine tRNA. *rns* and *rnl* are the small and large subunit ribosomal RNA genes, respectively. Genes are not drawn according to scale. (B) Maximum-likelihood tree derived from analysis of six concatenated mitochondrial genome-encoded proteins (Nad2, Nad3, Nad4, Nad6, Nad9, and Rps10). The clade containing eukaryotes is shaded. The analysis was based on 1137 unambiguously aligned positions. The tree was rooted arbitrarily with *Dehalococcoides* and Cyanobacteria. Numbers at nodes represent ML bootstrap values calculated with IQPNNI. Black dots on branches indicate bootstrap values ≥90%. Values below 50% are not shown.

Hydrogenosomes of well-studied groups such as trichomonads and fungi completely lack organellar genomes [10]. However, a hydrogenosome with a mitochondrial genome was recently described in the anaerobic ciliate *Nyctotherus* [9], further bolstering the evidence for a common evolutionary ancestry for both organelles [5, 10]. DNA-staining methods previously hinted that the *Blastocystis* MLOs also possessed a genome [3, 13]. By using three different strains, we confirmed this prediction by characterizing ∼6 kb segments of the organellar genome (Figure 2A), each of which encodes full-length mitochondrial-type small subunit (SSU) and large subunit (LSU) rRNAs, several tRNAs, and two NADH dehydrogenase (complex I) subunits of the ETC (discussed later). Furthermore, we recovered ESTs likely to be derived from the mitochondrial genome based on their distinctively low average G + C content of 20.4% (similar to the mean mitochondrial genome G + C content of 18.3% versus the mean nuclear G + C content of 60.33%). These ESTs encode three other mitochondrial NADH dehydrogenase (*nad*) subunits and a ribosomal protein. Protein sequences from all five *nad* subunits and ribosomal protein S10 were aligned with mitochondrial and eubacterial homologs and concatenated for phylogenetic analysis. In the maximum likelihood tree (Figure 2B), *Blastocystis* clusters weakly with other stramenopiles (bootstrap proportion [BP] = 52%) but within a mitochondrial clade that emerges next to *Rickettsia* and other α-proteobacteria with strong bootstrap support (BP = 100%). This pattern indicates that *Blastocystis* has a genome that is specifically related to other mitochondrial genomes that are descended from the chromosome of the ancestral endosymbiotic α-proteobacterium [5, 10]. This phylogenetic evidence; the close physical linkage of mitochondrial-type ribosomal RNA, tRNA, and *nad* genes (typically found only in mitochondrial DNA); the distinctively nonnuclear G + C content of these sequences; plus the demonstration of organellar DNA (Figure 1) all lead us to conclude that we have identified segments of a mitochondrial genome residing in the *Blastocystis* MLOs.

### *Blastocystis* Organelles Also Have Mitochondrial Energy Metabolism

**Figure 3:**
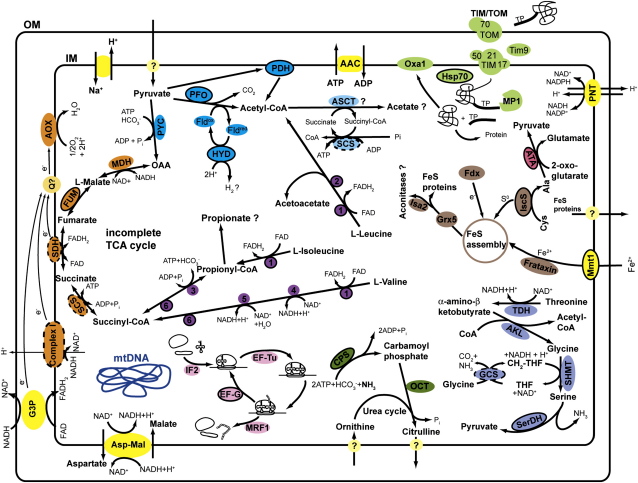
Proposed Metabolic Map of the *Blastocystis* Mitochondrion-like Organelles Proteins with predicted leader peptides have solid black outlines and protein complexes for which leader peptides for only some of the subunits have been predicted have dashed black outlines. The blue pathway represents the conversion of pyruvate to acetyl-CoA via the pyruvate dehydrogenase complex (PDH) or pyruvate:ferredoxin oxidoreductase (PFO). PFO reduces flavodoxin (Fld) and the [FeFe] hydrogenase (HYD) oxidizes flavodoxin with the concomitant production of molecular hydrogen; the question mark indicates that hydrogen production has not been demonstrated for *Blastocystis*. Acetyl-CoA is converted by the acetate:succinate CoA transferase (ASCT) to acetate (acetate production has not been tested for *Blastocystis*, but we identified an acetyl-CoA hydrolase like that known to function as ASCT in *Trichomonas* hydrogenosomes [35]). Transporters and translocators are shown in yellow. Components of the ETC and the partial TCA cycle are orange. Proteins involved in DNA transcription and translation are light pink. The mitochondrial part of the urea cycle is green. Pathways for the metabolism of the amino acids leucine, valine, and isoleucine are purple. Protein import and folding are light green. Pathways of the iron-sulfur cluster assembly are depicted in brown. Light blue represents the glycine-cleavage-system pathway. The question mark next to propionate shows that propionate production has not been assayed for *Blastocystis*; propionate is a metabolic end product of succinate degradation. For a complete list of abbreviations and numbers used in this figure see the Supplemental Data.

The presence of a mitochondrial genome coding for typical mitochondrial ETC components in the *Blastocystis* MLOs hints at mitochondrial-like biochemical properties, whereas the probable localization of [FeFe] hydrogenase and PFO in the same organelles is more suggestive of hydrogenosomal metabolism. To clarify the metabolic properties of the *Blastocystis* MLOs, we used 3330 clusters constructed from our EST data for a comparative BLAST search against the yeast and human mitochondrial proteomes [14, 15]. In addition, the KEGG Automatic Annotation Server was used to generate KEGG pathways. After manual curation, this analysis revealed 110 potential mitochondrion-targeted proteins and three putative mitochondrial genome-encoded proteins (collectively these represent 10.9% of all ESTs characterized), encompassing a wide variety of mitochondrial pathways (Table 1; see Table S2 for a full list of proteins). Of these proteins, 51 have a complete N terminus and are likely nuclear encoded, and mitochondrion-targeting peptides were predicted to be present on 34 of these proteins by at least two of four different prediction algorithms (see Figure 3 and Tables S1 and S2). A graphical overview of the predicted organellar metabolism with the major biochemical pathways identified through our BLAST comparisons and KEGG searches is shown in Figure 3, and below we discuss several in detail.

An important difference between the *Blastocystis* MLOs and other known hydrogenosomes and mitosomes is the presence of the multienzyme PDH complex (Figure 3 and Table S2). Thus, *Blastocystis* has two ways to decarboxylate pyruvate to form acetyl-CoA: one involves the classic mitochondrial PDH complex and the other involves the anaerobic PFO (Figure 3). Interestingly, although no PFO has been found yet in *Nyctotherus*, its organelles do seem to have a PDH complex, in contrast to the *Trichomonas* hydrogenosomes that have only PFO [9, 16–18]. *Euglena* and *Chlamydomonas* are the only other eukaryotes known to have both systems (aerobic PDH complex and anaerobic PFO/PNO), which enable the organisms to adapt to a broad range of oxygen levels in the environment [19, 20]. Whether the two systems serve a similar adaptive mechanism in *Blastocystis* is questionable because it is a strict anaerobe, and further biochemical testing is needed to help clarify this issue.

In typical mitochondrial metabolism, acetyl-CoA generated by the PDH complex feeds into the TCA cycle, which, in turn, generates reducing equivalents that drive oxidative phosphorylation. Curiously, in *Blastocystis* we have, thus far, only found a functionally interconnected subset of the enzymes of the TCA cycle (Figure 3 and Table S2). No ESTs were found corresponding to mitochondrial citrate synthase, aconitase, isocitrate dehydrogenase, or α-ketoglutarate dehydrogenase, and the absence of at least the latter two enzymes in *Blastocystis* is consistent with previous biochemical assays [4] that failed to detect these activities. Interestingly, the enzymes of the TCA cycle that we did find are known to function in the reverse direction as part of the branched pathway of malate dismutation in anaerobic metazoan mitochondria whereby malate is reduced to succinate, which is further metabolized to propionate in some organisms [8, 21]. However, that pathway is often coupled to oxidative phosphorylation via mitochondrial F_1_F_0_ ATPases, proteins that we were unable to identify among the *Blastocystis* ESTs (see below). Further biochemical testing will be required to determine the predominant direction of these pathways in *Blastocystis*. Although a similar incomplete TCA cycle also may exist in *Nyctotherus* [17], neither the *Trichomonas* hydrogenosomes nor any of the mitosomes described so far appear to have either a complete or a partial TCA cycle [10].

*Blastocystis* MLOs likely have complex I of the mitochondrial ETC, as nine subunits of the mitochondrial complex I were found among the ESTs, three of which are likely organelle encoded. Thus, including the two subunits encoded by the mitochondrial genome fragments, we have identified a total of 11 subunits out of the 42 described for human complex I [14]: six subunits involved in electron transfer from NADH to quinone and five subunits involved in proton pumping (see Table S2 and [22]). All four subunits of mitochondrial complex II (succinate dehydrogenase) were potentially identified (discussed below). In agreement with earlier biochemical studies [2, 4, 23], we found no evidence for the presence of complexes III and IV or an F_1_F_0_ ATPase in our EST data. Therefore, it is likely that the transmembrane potential of the *Blastocystis* MLOs, as shown with MitoLight and Rhodamine 123 staining [3, 4] and MitoTracker (Figure 1), results from a proton gradient generated solely by the activity of complex I.

Although mitochondrial cytochrome *c* oxidase (complex IV) usually passes electrons to oxygen as the terminal electron acceptor of the ETC, an alternative pathway has been described that involves an alternative oxidase (AOX) [24]. Electrons from complexes I and II are transferred to the AOX, which reduces oxygen to water but without proton translocation or subsequent energy production [25]. Activity of AOX depends on substrate and cofactor availability, such as quinone concentration, the redox state of quinone, and oxygen concentration [25, 26]. Interestingly we identified an AOX in *Blastocystis*. AOX also has been found in the mitosome-bearing apicomplexan *Cryptosporidium*, and the authors proposed that this could be the result of an evolutionary adaptation to oxygen stress conditions in the host intestine, which might be true for *Blastocystis* as well [27]. In addition to playing a role in redox balance, the AOX would be involved in preventing the formation of reactive oxygen species [24, 26].

Our findings suggest that the *Blastocystis* ETC is likely a true proton-pumping ETC similar to those in mitochondria and the organelles from the ciliate *Nyctotherus.* In the latter organism complex I appears to pass electrons via rhodoquinone to a fumarate reductase [8, 9, 17], which utilizes fumarate as a terminal electron acceptor converting it to succinate, as described for anaerobic mitochondria [8, 17]. Fumarate reductase and succinate dehydrogenase (i.e., complex II) are homologous enzymes that catalyze the same reaction but in reverse directions; within eukaryotes, these two enzymes are not clearly distinguishable at the sequence level [8, 21]. Thus, the subunits of complex II (SDH) we identified in *Blastocystis* could actually be a fumarate reductase. It is also possible that both enzyme activities could occur within the organelles at different times depending on the metabolic state of the cell.

Energy-producing organelles need to be able to export the ATP they produce into the surrounding cytosol. In mitochondria and hydrogenosomes this is accomplished by an ADP/ATP carrier (AAC), a unique eukaryotic protein with no homologs in prokaryotes. The *Trichomonas* hydrogenosomal HMP31 carrier has been termed an alternative AAC because it is not closely related to mitochondrial AAC proteins in phylogenetic trees, unlike other hydrogenosomal AACs [28]. We have identified several ESTs from *Blastocystis* that encode proteins with similarity to a mitochondrial carrier involved in adenosine translocation. At this point we assume that these represent organellar AACs; however, further testing is needed to corroborate our view.

### Other Essential Mitochondrial Functions

One of the essential functions of mitochondria is the assembly of FeS clusters [6, 29]. Evidence exists suggesting that proteins for this pathway are not only found in hydrogenosomes but also in mitosomes; indeed, FeS cluster assembly might be the only function of some mitosomes [6, 29, 30]. Our *Blastocystis* EST data revealed five proteins involved in FeS cluster assembly, about half of the proteins described for the yeast FeS assembly machinery [31]. No proteins involved in FeS cluster assembly have been found yet in the ciliate *Nyctotherus*, which might be explained by the relatively small number (2000) of genomic DNA clones analyzed [9, 17].

Organelles require an import machinery to transport nuclear encoded proteins into the organelle. Some variant of the TIM/TOM protein import mechanism seems to be common to all types of mitochondrion-related organelles, although probably with different levels of complexity [10, 30, 32]. We identified several proteins of the TIM/TOM import machinery in *Blastocystis* (Figure 3 and Table S2), and although we could not find a mitochondrial-processing peptidase (responsible for cleavage of leader peptides after import), we identified a metalloprotease 1 protein that might have the same function (Figure 3 and Table S2).

The presence of urea-cycle enzymes in the *Blastocystis* MLOs also is remarkable because, with the exception of the chytridiomycete *Neocallimastix* [33], none of the well-characterized hydrogenosomes or mitosomes are known to contain these proteins [5, 6]. It is possible that some of these enzymes instead function in a cytosolic arginine dihydrolase (ADH) pathway, where arginine is degraded to ammonia with the formation of ATP. However, a carbamate kinase was absent among the ESTs, the enzyme involved in the final ATP-forming step of the ADH pathway. In any case one of the enzymes we have identified, carbamoyl phosphate synthase (CPS), does have a predicted mitochondrial targeting peptide (Figure 3).

### The Evolutionary Transition between Mitochondria and Hydrogenosomes

Because *Blastocystis* and *Nyctotherus* are not closely related, their organelles represent a striking case of convergent evolution driven by adaptation to oxygen-poor environments. Both have retained a number of mitochondrial proteins and an organellar genome (Table 1). Strikingly, both appear to have lost many of the same components of the ETC (complexes III and IV) and, possibly, the ability to make ATP by oxidative phosphorylation. At the same time, both seem to contain organelle-targeted enzymes characteristic of anaerobic hydrogenosomal metabolism. The genes encoding hydrogenosomal enzymes are either ancestral to all eukaryotes and have been widely lost in aerobic lineages or they have been circulated among anaerobic eukaryotes by multiple events of lateral gene transfer [5].

We propose that the organelles of *Blastocystis* evolved from aerobically respiring mitochondria in adaptation to its extreme anaerobic lifestyle. These organelles, like those of *Nyctotherus*, apparently possess pathways typical for aerobic and anaerobic mitochondria as well as those for hydrogenosomes [10]. The discrete categories of mitochondria, hydrogenosomes, and mitosomes are, thus, insufficient to describe the evolutionary continuum that exists between these organelle types. *Blastocystis*, unlike *Nyctotherus*, can be grown axenically and is an ideal model organism in which to study these unique anaerobic organelles.

## Experimental Procedures

Total RNA from strain NandII was used for library construction by Amplicon Express (Washington). Complementary DNAs (cDNAs) were sequenced from the 5′ end to generate ESTs and where necessary 5′ and 3′ rapid amplification of cDNA ends (RACE) was performed to obtain full-length genes. ESTs were assembled into clusters and incorporated into the TBestDB database [34]. Three strains of *Blastocystis* were used for DNA isolation: NandII, BT-1, and DMP/02-328. Amplification of portions of the organellar genome was carried out for all three strains. SSU ribosomal RNA gene sequences obtained by PCR were used to construct specific primers, which were then employed in PCR-based primer walking along the genome. Phylogenetic analyses were carried out after alignment of the sequences by using ClustalX followed by manual editing in which ambiguously aligned sites were excluded. All organellar genome-encoded protein sequences were used in a concatenated analysis for phylogenetic reconstruction. Trees were calculated by using maximum-likelihood (ML) and Bayesian methods. ML bootstrap analyses were derived to estimate statistical support for each node. A homologous antibody raised against the *Blastocystis* [FeFe] hydrogenase was used in confocal immunofluorescence microscopy for localization of the protein within cells. DAPI and MitoTracker staining were carried out to visualize DNA and organelles, and as a negative control cells were stained with CellTracker. An in silico analysis of all ESTs sequenced from the cDNA library was carried out to classify the transcripts as orthologs of human/yeast mitochondrial or nuclear/cytosolic proteomes. In addition, the KEGG Automatic Annotation Server was used to generate KEGG pathways. A more detailed description of all strain material and methods used is provided in the Supplemental Data.

## Figures and Tables

**Table 1:** Comparison of Major Metabolic Properties of Mitochondria, Hydrogenosomes, and Mitosomes

Property	*Homo*	*Blastocystis*	*Nyctotherus*	*Trichomonas*	*Giardia*	*Encephalitozoon*
Organelle type	M [14]	MLO	H [9, 17]	H [36]	MS [30]	MS [37]
Organellar genome	+	+	+	−	−	−
Organellar genome maintenance proteins	+	+	+	−	−	−
Complex I^a^	≥42	11^b^	7	2	−	−
Complex II^a^	4	4^c^	2^c^	−	−	−
Complex III/IV	+	?	?	−	−	−
ATPase	+	?	?	−	−	−
AOX	−	+	?	−	−	−
PFO	−	+	?	+	+ (cyt)	−
PDH	+	+	+	−	−	+^d^
[FeFe] Hydrogenase	−	+	+	+	+ (cyt)	−
Fe-S assembly	+	+	?	+	+	+
TCA cycle	+	incomplete	incomplete	−	−	−
AA metabolism	+	+	+	+	−	−
Protein import and folding	+	+	+	+	+	+
Fatty acid metabolism	+	+	+	−	−	−
Carrier proteins^e^	47 (+)	9 (+)	2 (+)	5 (HMP31)	−	−

Abbreviations: M, mitochondrion; MLO, mitochondrion-like organelle; H, hydrogenosome; MS, mitosome; ATPase, mitochondrial ATPase; AOX, alternative oxidase; PFO, pyruvate:ferredoxin oxidoreductase; PDH, pyruvate dehydrogenase complex; Fe-S, iron sulfur cluster; TCA, tricarboxylic acid; AA, amino acid; ?, no evidence from EST data or biochemical studies; +, presence as determined by EST data and/or biochemical studies; −, absence as determined by whole-genome sequence data and/or biochemical studies; and cyt, cytoplasmic.

a Complex I and II each consist of several subunits.

b For *Blastocystis* we have identified 6 nuclear and 5 mitochondrial encoded subunits.

c For *Blastocystis* complex II could be a fumarate reductase; for *Nyctotherus* complex II has been annotated as fumarate reductase.

d Only the PDH E1 subunit has been described for *Encephalitozoon*.

e Number of mitochondrial carrier proteins including an AAC (ADP/ATP carrier) (+); for *Trichomonas* the AAC carrier is HMP31.

## Accession Numbers

All data generated in this study have been deposited in GenBank under the following accession numbers: EF494738–EF494740EF494738EF494739EF494740, EF512299–EF512302EF512299EF512300EF512301EF512302, and EF571321. The GenBank accession numbers of the nuclear small subunit ribosomal gene sequences from *Blastocystis* sp. BT-1 and DMP/02-328 are EF494741 and EF494742, respectively. Additional accession numbers for all ESTs reported here are shown in Table S2.

## Supplemental Data

Document S1. Supplemental Experimental Procedures, Three Figures, and Two Tables

Table S1. Sequences of N-Terminal Leader Peptides for *Blastocystis* Putative Mitochondrial Proteins

Table S2. List of 113 Potential *Blastocystis* Mitochondrial Proteins and Proteins Associated with Mitochondria with Their EST Accession Numbers
