# Supplementary material for: Organelles in Blastocystis that Blur the Distinction between Mitochondria and Hydrogenosomes
Source: Curr Biol. 2008 Apr 22;18(8):580–5. doi: 10.1016/j.cub.2008.03.037 (PMC2428068; doi:10.1016/j.cub.2008.03.037)
Supplement: Document S1. Supplemental Experimental Procedures, Three Figures, and Two Tables [file mmc1.pdf]

# Supplemental Data



# Organelles in *Blastocystis* that Blur the Distinction between Mitochondria and Hydrogenosomes

**Alexandra Stechmann, Karleigh Hamblin, Vicente Pérez-Brocal, Daniel Gaston, Gregory S. Richmond, Mark van der Giezen, C. Graham Clark, and Andrew J. Roger**

## Supplemental Experimental Procedures

### Strain Origin(s)

*Blastocystis* sp. NandII was obtained from the American Type Culture Collection (ATCC 50177) and DNA from *Blastocystis* sp. BT-1 was obtained from the same source (ATCC 50608D). *Blastocystis* sp. NandII was grown at 36°C in anaerobic chambers on inspissated whole egg overlaid with Locke's solution as described [S1]. Human *Blastocystis* sp. DMP/02-328 was obtained during routine screening and was grown at 36°C with a mixed bacterial flora in LYSGM with 5% adult bovine serum. LYSGM is a modification of TYSGM-9 in which the trypticase and yeast extract of the latter are replaced with 0.25% yeast extract (Sigma) and 0.05% neutralized liver digest (Oxoid). Total DNA was obtained from *Blastocystis* sp. DMP/02-328 by using the PUREGENE DNA Purification Kit (Gentra Systems). Subtyping of *Blastocystis* sp. BT-1 and DMP/02-328 indicated that both strains are of subtype 4 [S2], whereas *Blastocystis* sp. NandII is subtype 1. The GenBank accession numbers of the nuclear small subunit ribosomal gene sequences from *Blastocystis* sp. BT-1 and DMP/02-328 are EF494741 and EF494742, respectively.

### RNA Isolation and Library Construction

Total RNA from *Blastocystis* sp. NandII was extracted with TRIzol Reagent (Invitrogen). Library construction was carried out by Amplicon Express (Washington); cDNAs were cloned into pBluescript SKII(+) (Stratagene) and grown in *E. coli* DH10b (Invitrogen). All ESTs were sequenced with the T3 sequencing primer. For end sequencing of ESTs, cDNA was created by using the Gene Racer kit (Invitrogen), and 5′ and 3′ RACE PCR was carried out where necessary.

### mtDNA Sequencing

The organellar DNA was obtained in the following way. Initially, part of the small subunit rRNA gene (*rns*) was amplified by PCR using primers based on conserved stramenopile mtDNA *rns* sequences and total DNA isolated from the axenic *Blastocystis* sp. NandII and BT-1. Analyses of the resulting sequences confirmed their mitochondrial origins. Total DNA was then digested with blunt-end-producing restriction enzymes and ligated to an adaptor. PCRs were carried out by using the adaptor-ligated DNA and primers specific for the *rns* gene and adaptor sequence. Products were cloned and sequenced. This anchored PCR process was repeated with new specific primers and new ligations to "walk" along the mtDNA.

The sequences obtained for NandII and BT-1 were reconfirmed, and the corresponding region of DMP/02-328 mtDNA was obtained by amplification of the entire mtDNA segment from total DNA. Direct sequencing of PCR products was performed on an ABI3730 with ABI Prism BigDye Terminator v3.0 reagents (Applied Biosystems). Several NandII EST sequences encoding portions of the mitochondrial large subunit ribosomal RNA (*rnl*) (EC649709, EC650434, EC650222, EC641578), identified by BLAST searches, were used to design a conserved reverse primer that allowed the amplification of this region of the gene and extended the sequences ~800 bp at the 3′ end in all three isolates. Similarly, the 5′ sequence from isolate BT-1 was used to design a primer that allowed us to gain ~750 additional bp of sequence toward the 5′ end in NandII.

BLAST searches against other stramenopile genomes were used to identify protein coding and structural RNA genes. tRNAs were predicted by tRNAscan-SE (http://www.genetics.wustl.edu/eddy/tRNAscan-SE/). G + C content was calculated with the program Generunner (v3.05). Alignments among the three isolates as well as with orthologous genes from other

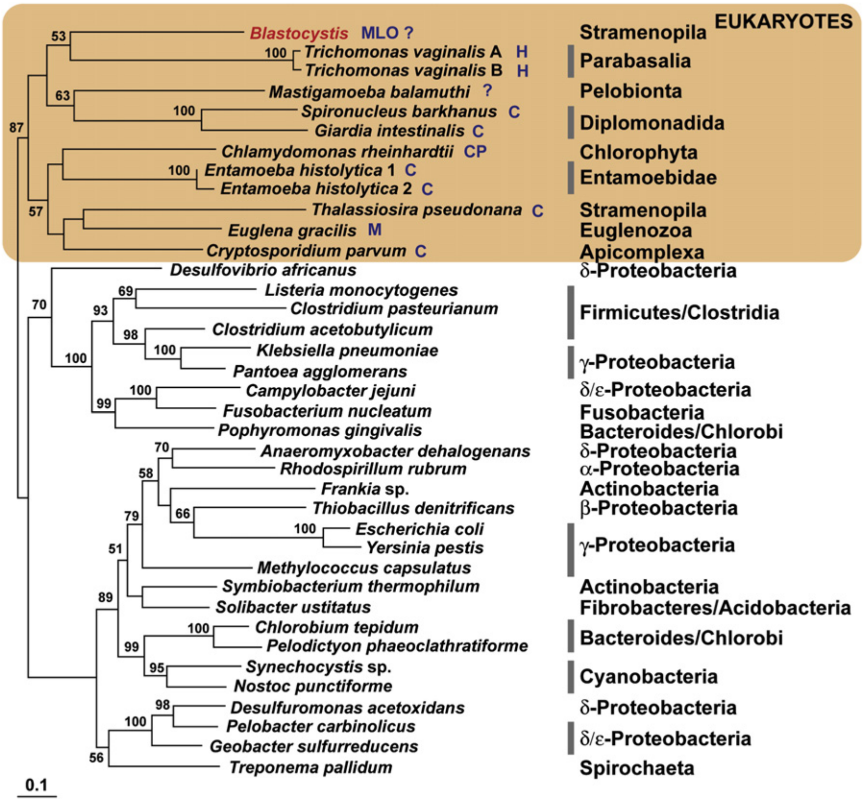


Figure S1. Maximum-Likelihood Tree of Eubacterial and Eukaryotic PFO Proteins

All eukaryotes form a monophyletic group, and the eukaryote clade is shaded. An α-proteobacterial ancestry of the eukaryote PFOs is not supported. Multiple lateral gene transfer events obscure the true phylogenetic pattern in the bacterial part. The *Blastocystis* sequence clusters weakly with proteins targeted into the Trichomonas hydrogenosome. Note that the *Euglena*, *Cryptosporidium*, and *Thalassiosira* proteins are part of a fusion protein composed of an N-terminal PFO and a C-terminal cytochrome P450-NADP oxidoreductase (PNO). The cellular localization for the eukaryotic PFOs is indicated in blue letters where known: MLO?, possible localization in the mitochondrion-like organelle; H, hydrogenosome; C, cytosolic; CP: chloroplast; M, mitochondrion; and ?, unknown. Numbers at nodes represent ML bootstrap values calculated with IQPNNI. The tree topology from the Bayesian analysis was congruent to the one presented here (not shown).

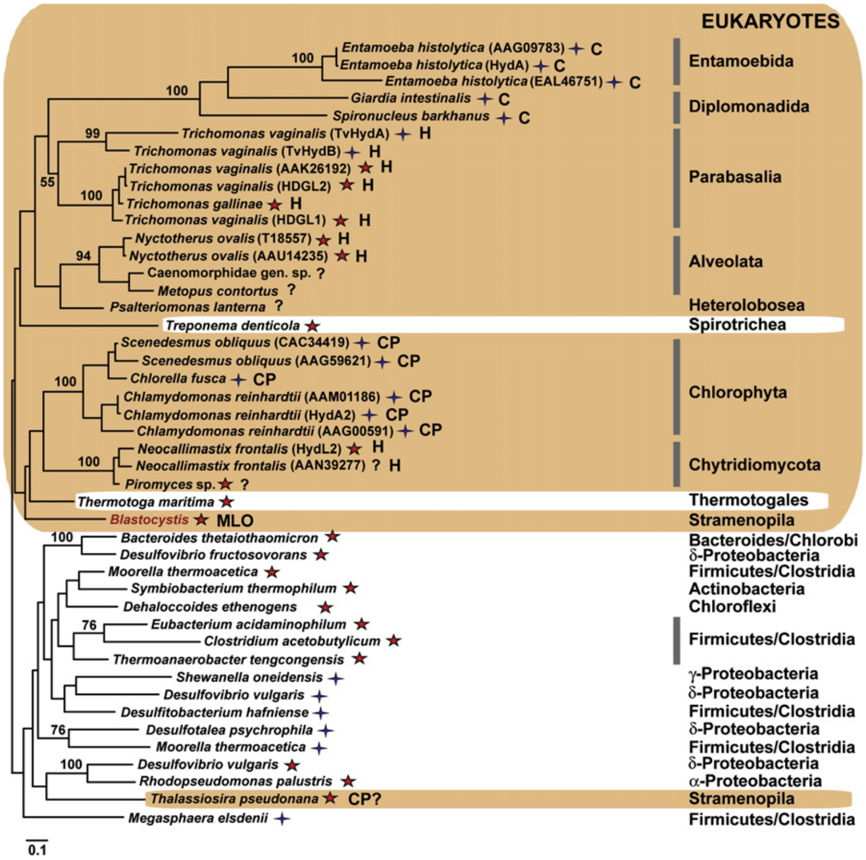


Figure S2. Maximum-Likelihood Tree of Eubacterial and Eukaryotic [FeFe] Hydrogenases

Archaebacterial genomes do not possess a gene encoding [FeFe] hydrogenase. Eukaryotes are shaded. The "long" hydrogenases are marked with red asterisks, the short ones with blue crosses; a question mark (?) is next to those in which the N-terminal part of the protein is missing and a classification into long or short, therefore, is not possible. The eukaryotic hydrogenases resolve into several poorly supported clades, and the *Blastocystis* hydrogenase shows no affinity for the other stramenopile sequence available, that of the free-living diatom *Thalassiosira*. The hydrogenosomal hydrogenases seem to be polyphyletic in origin; however, possible lateral gene transfer scenarios cannot be excluded. The bacterial hydrogenases show a similar pattern. The cellular localization of the protein is indicated where known: MLO, mitochondrion-like organelle; C, cytosolic; H, hydrogenosomal; CP, chloroplast; and CP?, probably chloroplast. For several proteins the N terminus is missing, and no information about the cellular localization is available (Caenomorphidae, *Metopus*, *Psalteriomonas*, *Piromyces*). The *Neocallimastix frontalis* AAN39277 protein is assumed to be hydrogenosomal. Accession numbers are indicated to distinguish between different copies of [FeFe] hydrogenases in one organism. Numbers at nodes represent ML bootstrap values calculated with IQPNNI. Bayesian analysis yielded a similar tree topology to the one presented here (not shown).

stramenopiles were conducted by using ClustalW implemented in the MEGA3 package [S3]. The GenBank accession numbers of the *Blastocystis* sp. BT-1, DMP/02-328, and NandII mt DNA regions generated in the present work are EF494738, EF494739, and EF494740, respectively.

### Phylogenetic Analyses

Additional [FeFe] hydrogenase, PFO, Nad, and Rps10 sequences were obtained from GenBank. Initial alignments were done by using ClustalX and then manually refined. The Nad3 protein in the alignment is from the BT-1 strain, and all other mtDNA-encoded proteins are from the NandII strain. All five Nad proteins and the Rps10 protein were concatenated to create a single alignment. Missing positions in cases of incomplete gene sequences or where genes were not present on the mitochondrial genome in some organisms were replaced with X. Regions of ambiguous homology were excluded before further analyses. Trees were constructed by using maximum likelihood (IQPNNI) and Bayesian inference (Mr.Bayes) [S4, S5]. The ML program used the WAG model for amino acid substitution and allowed for rate variation using a Gamma model with eight categories. Bootstrap analyses were done with 100 resamplings to estimate statistical support for branches in the optimal tree. Mr.Bayes312 was run with $10^6$ Markov Chain Monte Carlo generations by using a WAG model and eight gamma rates to allow for rate variation across sites. Four simultaneous chains were run and convergence was confirmed by manual inspection of the likelihood profiles. Trees from the first $10^5$ generations were discarded as burn-in. The statistical support for each node was assessed by posterior probabilities.

### Production of Recombinant Protein and Generation of Antibodies against [FeFe] Hydrogenase

The [FeFe] hydrogenase gene from *Blastocystis* sp. NandII was cloned into the pET-14b expression vector (Merck Biosciences) and then expressed in *E. coli* strain BL21pLysS. The His-tagged protein was purified on a nickel-chelating sepharose column and used for antibody production in rats (Eurogentec). The specificity of the *Blastocystis* [FeFe] hydrogenase antibody anti-*Blastocystis*Hyd was tested on a *Blastocystis* whole-cell lysate (strain DMP/02-328) using Western blots, where a band of 59 kDa could be detected (a size similar to the estimated molecular weight of the *Blastocystis* [FeFe] hydrogenase). The antibody was then used for confocal microscopy. Cells from the *Blastocystis* sp. DMP/02-328 were stained with 500 nM MitoTracker orange (Invitrogen) for 30 min, washed, and fixed in 3.7% formaldehyde. For control experiments, cells were stained simultaneously with MitoTracker (same conditions as just described) and 5μM CellTracker. For the [FeFe] hydrogenase localization a 1:100 dilution of anti-*Blastocystis*Hyd and a 1:200 dilution of the secondary anti-rat antibody conjugated to AlexaFluor 488 (Invitrogen) was used. Nuclear and mitochondrial DNA was stained with 2 μg/ml DAPI (4',6-diamidino-2-phenylindole). Images were viewed with a Zeiss LSM 510 meta laser-scanning confocal microscope with a 63× objective (oil immersion) and a 1 Airy unit confocal pinhole. Images were captured with LSM510 PCM software.

### Comparative BLAST Searching and KEGG Annotation of the Blastocystis Clusters

Human and yeast "subtractive" databases, comprising only nonmitochondrial proteins, were created with BLAST by using the *Homo sapiens* and *Saccharomyces cerevisiae* mitoproteome sequences as queries to search the corresponding whole proteomes of these organisms. Mitochondrial protein sequences came from the human mitoproteome and the yeast Mitop2 database [S6, S7]. Sequences in the whole proteomes that matched mitoproteome sequences over the entire query length and that had at least 99% sequence identity were removed from the whole proteome dataset to generate the subtractive nonmitochondrial proteome database. The *Blastocystis* clusters were then compared to all four databases, human/yeast nonmitochondrial subtractive and human/yeast mitoproteome, by using BLAST. The top-scoring hits for each of the subtractive and mitoproteome databases for a given organism (human or yeast) were compared, and the query was annotated as being mitochondrial or nonmitochondrial based on which of the databases yielded a hit with a higher score. The results of the yeast and human scores were then compared and queries sorted based on agreement between the organismal searches into one of four categories: Both human and yeast top hits were from the mitoproteome, both human and yeast top hits were from the subtractive proteome, human and yeast top hits were from different databases, or there were no significant hits in

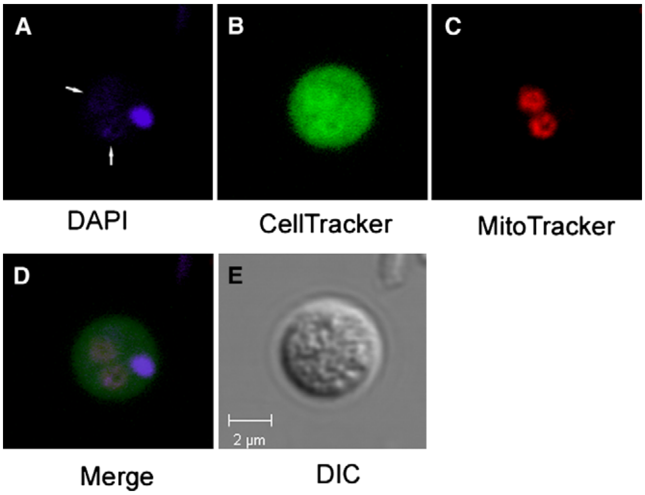


Figure S3. Control Experiment Showing Cytoplasmic and Organellar Staining of a *Blastocystis* Cell

A single *Blastocystis* cell with two mitochondrion-like organelles and one nucleus is shown. (A) DAPI staining of DNA in the organelles (arrows) and in the nucleus. (B) CellTracker staining of the cell cytoplasm. (C) Staining of the mitochondrion-like organelles with MitoTracker. (D) overlay of the DAPI, CellTracker, and MitoTracker staining. (E) DIC image of the *Blastocystis* cell.

Abbreviations for Figure 3: 1, branched chain amino acid transaminase; 2, isovaleryl-CoA dehydrogenase; 3, propionyl-CoA carboxylase; 4, 3-hydroxyisobutyrate dehydrogenase; 5, aldehyde dehydrogenase; 6, methylmalonyl-CoA mutase. AAC, ADP/ATP translocator; AKL, 2-amino-3-ketobutyrate coenzyme A ligase; AOX, alternative oxidase; ASCT, acetate-succinate CoA transferase; Asp-Mal, aspartate malate shuttle; ATA, alanine transaminase; CPS, carbamoyl phosphate synthase; EF-G, elongation factor G; EF-Tu, elongation factor Tu; Fdx, ferredoxin; Fld, flavodoxin; FUM, fumarate hydratase; G3P, glycerol-3-phosphate shuttle; GCS, glycine cleavage system with L-protein, T-protein, P-protein and H-protein; Grx5, glutaredoxin; Hsp70, mitochondrial 70kDa chaperone; IF2, translation initiation factor; IscS, cysteine desulfurase; Isa2, iron-binding IscA protein; MDH, malate dehydrogenase; Mmt1, putative iron transporter; MP1, metalloprotease 1; MRF1, peptide chain release factor; OCT, ornithine carbamoyl transferase; Oxa1, oxidase assembly-like protein; PNT, pyridine nucleotide transhydrogenase; PYC, pyruvate carboxylase; Q, quinone; SCS, succinyl-CoA synthetase; SDH, succinate dehydrogenase; SerDH, serine dehydrogenase; SHMT, serine hydroxymethyl transferase; TDH, threonine dehydrogenase; and TP, targeting peptide.

any of the databases. All potential mitochondrial proteins were then subjected to a final round of BLAST comparisons against the GenBank nonredundant database. In addition the *Blastocystis* ESTs were fed into the KEGG EGassembler server, and the output was used as a gene set for the KEGG Automatic Annotation Server with the bidirectional best hit method to assign orthologs. The KEGG Orthology assignments were used to automatically generate KEGG metabolic pathways.

### Supplemental References

S1. Zierdt, C.H., Donnolley, C.T., Muller, J., and Constantopoulos, G. (1988). Biochemical and ultrastructural study of *Blastocystis hominis*. J. Clin. Microbiol. *26*, 965–970.

S2. Stensvold, C.R., Suresh, G.K., Tan, K.S., Thompson, R.C., Traub, R.J., Viscogliosi, E., Yoshikawa, H., and Clark, C.G. (2007). Terminology for *Blastocystis* subtypes–a consensus. Trends Parasitol. *23*, 93–96.

S3. Kumar, S., Tamura, K., and Nei, M. (2004). MEGA3: Integrated software for Molecular Evolutionary Genetics Analysis and sequence alignment. Brief. Bioinform. *5*, 150–163.

S4. Minh, B.Q., Vinh, le, S., von Haeseler, A., and Schmidt, H.A. (2005). pIQPNNI: parallel reconstruction of large maximum likelihood phylogenies. Bioinformatics *21*, 3794–3796.

S5. Ronquist, F., and Huelsenbeck, J.P. (2003). MrBayes 3: Bayesian phylogenetic inference under mixed models. Bioinformatics *19*, 1572–1574.

S6. Taylor, S.W., Fahy, E., Zhang, B., Glenn, G.M., Warnock, D.E., Wiley, S., Murphy, A.N., Gaucher, S.P., Capaldi, R.A., Gibson, B.W., et al. (2003). Characterization of the human heart mitochondrial proteome. Nat. Biotechnol. *21*, 281–286.

S7. Andreoli, C., Prokisch, H., Hörtnagel, K., Mueller, J.C., Münsterkötter, M., Scharfe, C., and Meitinger, T. (2004). MitoP2, an integrated database on mitochondrial proteins in yeast and man. Nucleic Acids Res. *32*, D459–D462.
