# Supplementary material for: Organelles in Blastocystis that Blur the Distinction between Mitochondria and Hydrogenosomes
Source: Curr Biol. 2008 Apr 22;18(8):580–5. doi: 10.1016/j.cub.2008.03.037 (PMC2428068; doi:10.1016/j.cub.2008.03.037)
Supplement: Table S1. Sequences of N-Terminal Leader Peptides for Blastocystis Putative Mitochondrial Proteins [file mmc2.pdf]

SD Table 1: Sequences of N-terminal leader peptides for *Blastocystis* putative mitochondrial proteins

**protein**	**MitoProt**	**probability**
Cystein desulfurase (IscS)	MLSRFSSVIARAPAVLSLSCRALPNGIHHM	0.9688
Mitochondrial metal transporter (Mmt1)	MSFVVLRCSLARSPFQTAAIIRVASNRGLEACGKVRF	0.9784
Pyruvate dehydrogenase (PDH E1alpha)	MISRIAVKGIPRQFVRAFSSRTFTVELPEF	0.9866
Dihydrolipoyl dehydrogenase (PDH E3)	MLSLSRASVIAQRSLYAARFFSAAAADK	0.9398
Dihydrolipoyl transacetylase (PDH E2)	MLASKLARSVVNPKGAALLRHYCSDYTKHFLMPSAARLVALNHLDASNIVGTGLGGRIMRY	0.6775
Fumarase	MFALAKSAPKFAAASKVAVRGVATRT	0.9515
Succinyl-CoA synthetase subunit beta (SCS beta)	MLRMAPKTVGAVRNLNIHEWQSKQLIQKYGGRA	0.6301
Alanine transaminase	MLRLTKNVALPTLRCFSSRITID	0.9736
P-protein	MQSLLRQTLRRSSNAGMAIRCFTAARPFAPCFKFV	0.9963
T-protein	MLARFAQTLAKPTARAFSSGAAIA	0.9839
L-protein	MLSLSRASVIAQRSLYAARFFSAAAADK	0.9398
Serine dehydrogenase	MKRTSSHIRIISKKGNSH	0.8505
Branched chain amino acid transaminase	MMMSKSSVLARSFSAAKTNI	0.5349
Isovaleryl-CoA dehydratase	MLHSLCKQVGRCALSRYLPCVLHNPCFATLSKRAFSTDNECE	0.9559
Carbamoyl phosphate synthetase (CPS)	MFGRSFAGISSVSRIARSFATEAELP	0.9753
Methylmalonyl-CoA mutase	MLAVSRQLTKSSVRAFAVTPYPE	0.9697
mt Hsp70	MRSSLATLARTHMRT	0.6131
Glyoxalase I	MFANVAKRAFATGKPYRILGVQQIAI	0.9588
Acetyl-CoA carboxylase	MLSKRAVAMTRSFAGAVDLT	0.7623
Mitochondrial elongation factor Tu (EF-Tu)	MRLVAGKQMVPAFTSVRNATYERK	0.2431
Mitochondrial Ribosomal protein (MRP-L4)	MLSRCFGACKTVACSWIKTPRV	0.9786
Mitochondrial ribosomal protein (MRP-L22)	MSLLCRLGSVASIGRGISTASRVFSQNSLLGLPALMEMTNSPACMCCTQFIRSFSHPTKKY	0.7917
Mitochondrial ribosomal protein (MRP-L54/L37)	MQSLLKLASIPRAALRSGAILAPQWHAASVYASTLSPFTRSFASDKKKG	0.9866
Mitochondrial ADP/ATP carrier (AAC)	MGKKQDRRLTFMQNFIAGGIAGVGSRTFTSPLDVVKIIAQVGSKQHSGFIGTFKNIYKQEGLRGFWKGNGVACLRLFPYSAINF	0.7188
ETF:ubiquinone oxidoreductase	MFRSLQRAVKQSFPSLLFLRGRGYPSLSFFTRVLSTESTET	0.9945
Thioredoxin peroxidase	MLPKIAKTVASASKRFGGVSAFTQVARSFSQITYGPSQAFVRQ	0.9559
Thioredoxin	MVLPSVASRAGSMVFQRGFASAQDKV	0.8934
Pyruvate:ferredoxin oxidoreductase (PFO)	MFNTLVKRAMTSAARYNSVCAATLPKAVIARN	0.988
[FeFe] hydrogenase	MLSRLSRIATTKSMLVMNAARSFAAEAQGK	0.9304
Complex I, subunit NDUFA2	MLRCSSKQSRMRPRAEFGTRI	0.843
Complex I, subunit NDUFA5	MFATKALLATVKRT	0.4043
Complex I, subunit NDUFS7	MRRYTLTEGNMQRVISSAAKAA	0.8712
Complex I, subunit NDUFA9	MLARFANKPSVRVLARSITSDVFPGTLGLGGRMSQAPQKVTIFGASTGNLGRSVVYELASRGVTCMIPYRG	0.993
Succinate dehydrogenase flavoprotein A (SDH-A)	MLPSFRIMGKSAPALTRAFRATACRS	0.9627
Succinate dehydrogenase iron sulphur protein (SDH-ip)	MFARSLSSLSKVAVRGSVVLPQISAMNFSTGFKIGPDGKIPSYKLFSLFRY	0.9801

SP: secretory pathway
mtSP: mitochondrial targeting peptide
NO: no targeting peptide predicted

SD Table 1 continued

protein	TargetP	probability	SignalP	probability	iPSORT
Cystein desulfurase (IscS)	MLSRF	0.787	MLSRFSSVIARAPAVLSLSCRA	0.915	SP
Mitochondrial metal transporter (Mmt1)	MSFVVLRCSLARSPFQTAAIIRVASNRGLEACGKVRFM	0.905	MSFVVLRCSLARSPFQTAA	0.301	mtSP
Pyruvate dehydrogenase (PDH E1alpha)	MISRIAVKGIPRQFVRAFSSRTFTV	0.805	MISRIAVKGIPRQFVRAF		mtSP
Dihydrolipoyl dehydrogenase (PDH E3)	MLSLSRASVIAQRSLYAARFF	0.879	MLSLSRASVIAQRSLYAARFFSAAAA	0.462	NO
Dihydrolipoyl transacetylase (PDH E2)	MLASKLARSVVNPKGAALLRHY	0.875	NO		mtSP
Fumarase	MFALAKSAPKFAAASKVAVRG	0.612	MFALAKSAPKFAAASKVAVRGVAT	0.15	NO
Succinyl-CoA synthetase subunit beta (SCS beta)	MLRMAPKTVGAVRN	0.524	NO		mtSP
Alanine transaminase	MLRLTKNVALPTLRCF	0.743	NO		mtSP
P-protein	MQSLLRQTLRRSSNAGMAIRCF	0.952	NO		mtSP
T-protein	MLARFAQTLAKPTARAFSSGAAIA	0.677	MLARFAQTLAKPTARAFSSGAAIAHTPL	0.107	NO
L-protein	MLSLSRASVIAQRSLYAARFF	0.879	MLSLSRASVIAQRSLYAARFFSAAAA	0.462	NO
Serine dehydrogenase	NO		NO		mtSP
Branched chain amino acid transaminase	MMMSKSSVLARSFSAAKTNI	0.506	NO		mtSP
Isovaleryl-CoA dehydratase	MLHSLCKQVGRCALSRYLPCVLHNPCFATLSKRAF	0.756	NO		mtSP
Carbamoyl phosphate synthetase (CPS)	MFGRSFAGISSVSRIARSF	0.796	MFGRSFAGISSVSRIARSFA	0.467	mtSP
Methylmalonyl-CoA mutase	MLAVSRQLTKSSVRAFAVTPYPE	0.663	NO		mtSP
mt Hsp70	MRSSLATLARTHMRTM	0.498	MRSSLATLARTHMRTMATA	0.297	NO
Glyoxalase I	MFANVAKRAF	0.694	NO		mtSP
Acetyl-CoA carboxylase	MLSKRA	0.707	NO		NO
Mitochondrial elongation factor Tu (EF-Tu)	MRLVAGKQMVPAFTSVRN	0.566	NO		NO
Mitochondrial Ribosomal protein (MRP-L4)	MLSRCF	0.683	NO		mtSP
Mitochondrial ribosomal protein (MRP-L22)	MSLLCRLGSVASIGRGISTASRVFSQNSLLGLPALMEMTNSPACMCCTQFIRSF	0.917	MSLLCRLGSVASIGR	0.517	mtSP
Mitochondrial ribosomal protein (MRP-L54/L37)	MQSLLKLASIPRAALRSGAILAPQWHAASVYASTLSPFTRSF	0.804	MQSLLKLASIPRAALRSGAILAPQWHAASVY	0.874	mtSP
Mitochondrial ADP/ATP carrier (AAC)	MGKKQDRRLTFMQNFIAGGIAGVGSRTF	0.587	NO		NO
ETF:ubiquinone oxidoreductase	MFRSLQRA	0.955	probability for signal anchor	0.723	mtSP
Thioredoxin peroxidase	MLPKIAKTVASASKRFGGVSAFTQVARSFSQITYGP	0.881	MLPKIAKTVASASKRFGGVSAFTQVARSFS	0.342	mtSP
Thioredoxin	MVLPSVASRAGSMVFQRGF	0.561	MVLPSVASRAGSMVFQRGFASA	0.116	NO
Pyruvate:ferredoxin oxidoreductase (PFO)	MFNTLVKRAMTSAARYNSVCAATLPKAVIARN	0.863	MFNTLVKRAMTSAARYNSVCAATLPKAVIARN	0.111	NO
[FeFe] hydrogenase	MLSRLSRIATTKSMLVMNAARSF	0.831	MLSRLSRIATTKSMLVMNAARSFA	0.434	mtSP
Complex I, subunit NDUFA2	MLRCS	0.71	NO		mtSP
Complex I, subunit NDUFA5	MFATKALLATVKRTTGMVGLPVIPNARA	0.356	MFATKALLATVKRTTGMVGLPVIPNARA	0.492	NO
Complex I, subunit NDUFS7	MRRYTLTEGNMQRVISSAAKAASKNAIQTY	0.702	NO		mtSP
Complex I, subunit NDUFA9	MLARFANKPSVRVLARSITSDVFPGTLGLGGRMSQAPQKVTIFGASTGNLGRSVVYELASRG	0.731	NO	NO	mtSP
Succinate dehydrogenase flavoprotein A (SDH-A)	MLPSFRIMGKSAPALTRAFRATACRS	0.882	MLPSFRIMGKSAPALTRAFRATACRSAVVGSTADA	0.546	mtSP
Succinate dehydrogenase iron sulphur protein (SDH-ip)	MFARS	0.778	MFARSLSSLSKVAVRGSVVLPQISA	0.449	SP

SP: secretory pathway
mtSP: mitochondrial targeting peptide
NO: no targeting peptide predicted
