# Supplementary material for: Organelles in Blastocystis that Blur the Distinction between Mitochondria and Hydrogenosomes
Source: Curr Biol. 2008 Apr 22;18(8):580–5. doi: 10.1016/j.cub.2008.03.037 (PMC2428068; doi:10.1016/j.cub.2008.03.037)
Supplement: Table S2. List of 113 Potential Blastocystis Mitochondrial Proteins and Proteins Associated with Mitochondria with Their EST Accession Numbers [file mmc3.pdf]

Table 2. List of 113 potential *Blastocystis* mitochondrial proteins and proteins associated with mitochondria with their EST accession numbers

Metabolic pathway/component	Gene product	Gene function	Target Signal*	Accession no.
Complex I (protein annotation as for *Homo sapiens*)	NADH dehydrogenase subunit NDUFS8	removal of two electrons from NADH and transfer to ubiquinone coupled with moving 4 protons across the membrane to create a proton gradient	??	EC648261
	NADH dehydrogenase subunit NDUFA2		+	EC648357, EC647734, EC640111
	NADH dehydrogenase subunit NDUFA5		+	EC646670, EC642739, EC648955
	NADH dehydrogenase subunit NDUFS7		+	EC651574, EC645176, EC641240
	NADH dehydrogenase subunit NDUFA9		+	EC651599, EC649395, EC643171
	NADH dehydrogenase subunit NDUFV1		??	EC642144, EC649297, EC642104, EC641703, EC640678
	NADH dehydrogenase subunit ND4		orgDNA?	EF512301 †
	NADH dehydrogenase subunit ND2		orgDNA?	EF512302 †
	NADH dehydrogenase subunit NDUFS3 (=ND9)		orgDNA?	EF512302 †
Complex II (TCA cycle) or fumarate reductase	Succinate dehydrogenase flavoprotein A (SDH1-1, SDH1-2)	SDH oxidizes succinate into fumarate while passing electrons via FAD to ubiquinone, FRD: reduction of fumarate to succcinate	+	EC644724, EC645380, EC643838, EC650234, EC649122, EC649237, EC648601, EC648130, EC647212
	SDH iron sulphur protein (SDH-ip)		+	EC648965, EC648221, EC644666, EC646657
	SDH subunit C (CII-3), cytochrome b560		?	EC651608, EC645437, EC650062, EC648766, EC647940, EC646492, EC651129, EC641865, EC642714, EC641045
	SDH subunit D (QPs3), cytochrome b small subunit (also anchor protein)		?	EC644822, EC644015
AOX	Alternative oxidase	terminal electron acceptor	??	EC651344, EC650155, EC650078, EC649563
PNT	Pyridine nucleotide transhydrogenase	proton translocator	??	EC650696, EC641674, EC645828, EC642368
Anaerobic H_2-metabolism	[FeFe] hydrogenase	electron acceptor/donor	+	EF512299 †
	PFO	anaerobic decarboxylation of pyruvate to acetyl-CoA	+	EF512300 †

Fe-S cluster asssembly	Frataxin	putative iron donor	?	EC650610, EC642473
	[2Fe-2S] Ferredoxin (Fdx)	electron transfer protein	??	EC644462, EC644151
	Glutaredoxin (Grx5)	regulates redox state of cysteine residues	?	EC650607, EC646727
	Cysteine desulfurase (IscS)	sulphur donor	+	EC650805, EC650524
	putative ABC transporter ATPase (ycf16)	similarity to bacterial SufC	??	EC644813, EC641049
	mitochondrial metal transporter 1 (Mmt1)	involved in iron uptake	+	EC650311
	Iron sulphur assembly protein Isca2	probably involved in iron binding	??	EC641895
	putative ABC transporter	similarity to bacterial SufBD	??	EC649314
PDH multienzyme complex	Pyruvate dehydrogenase (PDH E1α)	decarboxylation of pyruvate with the formation of acetyl-CoA, CO_2 and NADH + H^+	+	EF571319
	Dihydrolipoyl transacetylase (PDH E2) ‡		+	EC650042, EC647928
	Dihydrolipoyl dehydrogenase (PDH E3) §		+	EF571320
Incomplete TCA cycle	Malate dehydrogenase (MDH)	catalyses conversion of malate into oxaloacetate and vice versa	??	EC639479, EC647720
	Fumarate hydratase (FUM)	catalyses conversion of fumarate to malate and vice versa	+	EC650252, EC646717, EC645175, EC643516, EC650299, EC648196, EC644896, EC644366, EC647875, EC646066, EC644739, EC643864, EC644693, EC642693, EC650931, EC644366
	Succinyl CoA synthase subunit alpha (probably ATP forming)	conversion of succinyl-CoA to succinate coupled with substrate level phosphorylation	-	EC649868, EC649367, EC649326, EC648512, EC650807, EC651453, EC649523, EC650157, EC649259, EC648970, EC648109, EC646528, EC646784, EC647694, EC646851
	Succinyl CoA synthase subunit beta (probably ATP forming)		+	EC648114, EC643655, EC642446, EC650994, EC649690, EC649221, EC647375, EC645507, EC644723, EC644096
AA metabolism	Aspartate aminotransferase (DTA)	amino group transfer	??	EC651540, EC648734, EC649876, EC648727, EC648489, EC647643, EC645975, EC651603, EC649364, EC648159, EC646989, EC649574, EC643094

	Alanine aminotransferase (ATA)	amino group transfer	+	EC651439, EC650650, EC650476, EC650372, EC650304, EC650569, EC650018, EC650055, EC649448, EC649047, EC648487
	Serine hydroxymethyltransferase (SHMT)	Threonine/Serine/Glycine degradation	??	EC646525, EC651022, EC649971, EC649343, EC649178, EC649124, EC648510, EC647923, EC648857, EC641155, EC644501, EC651327, EC650077, EC646207, EC645417, EC648697, EC647293, EC646685, EC648674, EC646562, EC645915
	P-protein (glycine dehydrogenase)	Glycine cleavage system	+	EC649856, EC645928, EC651548, EC647446, EC644683, EC644550
	T-protein (aminomethyltransferase)	Glycine cleavage system	+	EC646166, EC645811, EC640520, EC641964
	L-protein (dihydrolipoyl dehydrogenase) [§]	Glycine cleavage system	+	EC642260, EC641617, EC642462
	H-protein (aminomethyl carrier)	Glycine cleavage system	??	EC645562, EC644836, EC644514, EC645149
	2-amino-3-ketobutyrate coenzyme A ligase	Thr/Ser/Gly degradation	-	EC650438, EC650776, EC650547, EC650849, EC650083, EC649957, EC648986, EC649506, EC650270, EC649437, EC648292, EC649144, EC648388
	Serine dehydrogenase	catabolises serine to pyruvate	+	EC649883
	Threonine dehydrogenase	Threonine degradation	??	EC643109
	Branched chain amino acid transaminase	first step in degradation of branched chain amino acids	+	EC650634, EC644342, EC643312, EC642998, EC642344
	Isovaleryl-CoA dehydrogenase	L-Leucine degradation	+	EC648609
	Propionyl-CoA carboxylase (alpha chain)	L-Isoleucine degradation or malate dismutation	??	EC648774, EC649794, EC649499, EC650306, EC644996, EC644843, EC645178, EC648037, EC644017, EC643957, EC648218, EC647409, EC645432
	3-hydroxyisobutyrate dehydrogenase	L-Valine degradation	??	EC650629
	Methylmalonyl-CoA mutase	L-Valine and L-Isoleucine degradation or malate dismutation	+	EC644515, EC643690, EC648880, EC648382, EC648470, EC649492, EC646542, EC645960, EC643022

Protein import and folding	Hsp70	part of mitochondrial preprotein import apparatus	+	EC646843
	TOM 70	promotes substrate binding		EC648821, EC644043
	TIM 50	regulates channel opening		EC648841
	TIM 21[¶]	regulates module docking		EC649725, EC645410
	TIM17	translocation channel		EC648066
	TIM 9	transfer of substrates for TIM22		EC650533, EC646626
	Metalloprotease 1 (MP1)	probable processing peptidase	??	EC651375
	Oxidase assembly like protein (OXA1)	probably required for assembly of proteins in the inner membrane	-	EC649010
Pyruvate metabolism	Acetate:succinate CoA transferase (ASCT)	conversion of acetyl-CoA to acetate	??	EC651134, EC649637, EC649252, EC648431, EC646632, EC646798, EC646644, EC647239, EC643223
	Acetyl-CoA synthetase	acetate to acetyl-CoA conversion	??	EC649035
	Glyoxalase II	conversion of 2-oxoaldehyde into 2-hydroxy acids	-	EC643472, EC646079
	Lactoylglutathione lyase (glyoxalase I)	conversion of 2-oxoaldehyde into 2-hydroxy acids	+	EC650453
	Pyruvate carboxylase	produces OAA from pyruvate	??	EC639336, EC645309, EC644717, EC650808, EC648073, EC642631, EC642617, EC650219, EC650500, EC647549
Fatty acid metabolism	Enoyl-CoA hydratase	β-oxidation and/or mitochondrial fatty acid elongation	??	EC639510
	Trans-2-enoyl-CoA reductase (NADPH dependent)	mitochondrial fatty acid elongation	??	EC641413
	Aldehyde dehydrogenase	oxidation of aldehydes	SP	EC649910, EC649542, EC648575, EC645782
	Long chain fatty acid CoA ligase	β-oxidation	??	EC650632, EC639608, EC642825, EC648878, EC646624, EC645264, EC644773, EC644597, EC643542
	Acetyl-CoA carboxylase	transfer of phosphogroups	+	EC650624, EC650340, EC647974, EC646750, EC650236, EC641084, EC651111, EC649722, EC649548, EC649640, EC650577, EC649389, EC650488, EC648695, EC648820
	Malonyl-CoA ACP transacetylase (MAT)	fatty acid biosynthesis	?	EC649370

Proteins associated with mitochondrial translation	(MSD1) Aspartate tRNA ligase		??	EC642523
	Leucyl tRNA synthase		??	EC644262
	Elongation factor Tu		+	EC650363, EC650226, EC647761
	Translation initiation Ef-G		??	EC648584, EC642691
	Translation initiation factor IF-2		?	EF571321 [†]
	Transcription factor A		??	EC650837, EC649244, EC644913
	MRF1 – peptide chain release factor	recognizes UAA and UGA stop codons, translation termination	??	EC650000
	MSS1 – GTPase, proofreading	involved in mt tRNA modification	??	EC648810
	MTO1 – protein synthesis, proofreading	involved in mt tRNA modification	??	EC643540
	Ribosomal protein S10 (MRP-S10)		orgDNA?	EF512302 [†]
	Ribosomal protein S15 (MRP-S15)		??	EC640558
	Ribosomal protein L4 (MRP-L4)		+	EC646714, EC649129
	Ribosomal protein L13 (MRP-L13)		??	EC649559
	Ribosomal protein L15 (MRP-L15)		??	EC642912, EC639448
	Ribosomal protein L22 (MRP-L22)		+	EC650947, EC648051, EC650998
	Ribosomal protein S35 (MRP-S35)		??	EC644470
	Ribosomal protein L54/L37 (MRP-L54/L37)		+	EC644909
	Ribosomal protein L24 (MRP-L24)		??	EC645199, EC641408, EC639953
Mitochondrial transporters/carriers	Oxoglutarate/malate carrier (aspartate malate shuttle)		??	EC643604, EC648873, EC646830, EC647307, EC642122, EC641487, EC646571, EC639987
	Mitochondrial carrier triple repeat protein (Mcart1)		-	EC651508, EC650799, EC650535, EC648136, EC648012, EC647525, EC646057, EC645894, EC645779
	Aspartate/Glutamate carrier, Aralar (Citrin)		?	EC645628, EC650587, EC651187, EC651431, EC650974, EC650788, EC650530, EC650464
	Mitochondrial carrier [‖]	putative ADP/ATP translocator	+	EC651395, EC651258, EC651094, EC650984, EC650939, EC650428, EC650643, EC650035, EC649970, EC650120, EC645384

	Carnitine/acylcarnitine carrier	catalyses the translocation of long chain fatty acids across the IM	?	EC650853, EC650126, EC649180, EC648840, EC648676, EC648345, EC646725, EC646706, EC646314, EC644991
	Phosphate carrier	uptake of P_i and H^+	-	EC651456, EC651234, EC651166, EC650076, EC649454, EC649415, EC649797, EC649423, EC648276, EC648235, EC650589
	S-adenosylmethionine carrier protein		SP	EC645511
	Voltage dependent anion channel		??	EC644980, EC650620, EC647544
	NA^+/H^+ antiporter (only prokaryote hits)		??	EC644931
Others	Mitochondrial acyl carrier protein	cofactor	??	EC646526, EC648882
	Glycerol-3-phosphate dehydrogenase (GUT 2)	provides reducing equivalents for the ETC	??	EC646107, EC649054, EC651227, EC648107, EC645035, EC650185
	Electron transferring flavoprotein ubiquinone oxidoreductase	electron transfer protein	+	EC646109
	Electron transport flavoprotein, alpha polypeptide (Alpha-ETF)	electron transfer protein	??	EC644907
	Succinate semialdehyde dehydrogenase	GABA catabolism	??	EC647947
	Prohibitin – mito inner membrane	acts as chaperone to stabilize mito proteins	??	EC646500, EC648475, EC642422
	Adenylate kinase	2ADP = ATP + AMP	??	EC648046, EC642527
	Lipoic acid synthetase	lipoate biosynthesis	??	EC641444
	Lipoyltransferase (lipoate protein B ligase)	lipoate biosynthesis	??	EC646817, EC642471
	Pyruvate dehydrogenase kinase	inhibitory effect on pyruvate dehydrogenase complex	??	EC644318
	Sideroflexin 5	tricarboxylate carrier, potentio iron ion transporter	??	EC645183
	MSF1-protein	probable function in intramitochondrial protein sorting	-	EC651260, EC644847, EC642982
	AFG1	mitochondrial ATPase	??	EC646878, EC642847, EC642621, EC641021, EC645022
	Ornithine carbamoyltransferase (OTC)	urea cycle	??	EC648481, EC648125
	Carbamoyl phosphate synthase (CPS)	urea cycle	+	EC651070, EC651063, EC651062, EC651135, EC651472, EC648340, EC648071, EC647274, EC648168
	Tafazzin	acyl-specific transacylase	??	EC646053, EC643486

	Polypeptide deformylase (PDF)	involved in N-terminal methionine excision of mitochondrial proteins	??	EC649264
	Sirtuin 3	NAD-dependent deacetylase	??	EC650269
	Thioredoxin reductase	oxygen salvage	??	EC650904
	Thioredoxin peroxidase	oxygen salvage	+	EC651033, EC649953, EC644335
	Thioredoxin	oxygen salvage	+	EC644328, EC647268
Proteins from mt genome	NADH dehydrogenase subunit ND3		orgDNA	EF494738, EF494739, EF494740 †
	NADH dehydrogenase subunit ND6		orgDNA	EF494738, EF494739, EF494740 †
Proteins associated with mitochondria	Dynamin like protein	possibly involved in mitochondrial fission		EC650097, EC646754
	Ubiquinone biosynthesis protein			EC646586
	Phosphoenolpyruvate carboxykinase (PEPCK)	conversion between phosphoenolpyruvate and oxaloacetate		EC650958, EC648059, EC646693, EC640731

Description of symbols and abbreviations used: +, Presence of a signal peptide as predicted by two or more of all four analyses; -, Absence of a signal peptide despite full length N-terminus including a Met start codon; ?, Only one out of the four programs predicted an N-terminal signal peptide. ??, No full length N-terminus because no 5' Met available; orgDNA, organellar DNA; orgDNA?, possibly organellar DNA; SP, Sequence most probably contains a signal for the secretory pathway.

*: Target signal determined with TargetP, SignalP, iPSORT, and Mitoprot.

†: Genes for which full length sequences were obtained in this study and sequences generated by PCR in this study.

‡: Could also be E2 subunit of α-ketoglutarate dehydrogenase complex (succinyl transacetylase).

§: PDH E3 – homologous to L-protein of glycine cleavage system; possibly also α-ketoglutarate dehydrogenase complex E3 subunit.

¶: Very low Blast score for this protein.

||: Clusters show Blast similarities to Graves disease carrier (solute carrier family 25 member 16) and ADP/ATP translocator (solute carrier family 25 member 43).
